# A Non-Shedding Fruit *Elaeis oleifera* Palm Reveals Perturbations to Hormone Signaling, ROS Homeostasis, and Hemicellulose Metabolism

**DOI:** 10.3390/genes12111724

**Published:** 2021-10-28

**Authors:** Fabienne Morcillo, Julien Serret, Antoine Beckers, Myriam Collin, Sebastien Tisné, Simon George, Roberto Poveda, Claude Louise, Timothy John Tranbarger

**Affiliations:** 1 DIADE (Diversité, Adaptation, Développement des Plantes), University of Montpellier, CIRAD (Centre de Coopération Internationale en Recherche Agronomique pour le Développement), IRD (Institut de Recherche pour le Développement), 34393 Montpellier, France; fabienne.morcillo@cirad.fr (F.M.); julien.serret@ird.fr (J.S.); antoine.beckers31@gmail.com (A.B.); myriam.collin@ird.fr (M.C.); 2 CIRAD, UMR (Unité Mixte de Recherche) DIADE, 34398 Montpellier, France; 3 CIRAD, UMR AGAP (Amélioration Génétique et Adaptation des Plantes Méditerranéennes et Tropicales), 34398 Montpellier, France; sebastien.tisne@cirad.fr; 4 AGAP, University of Montpellier, CIRAD, INRAE (Institut National de Recherche pour l’Agriculture, l’Alimentation et l’Environnement), Institut Agro, 34398 Montpellier, France; 5 MGX-Montpellier GenomiX, University of Montpellier, CNRS (Centre National de la Recherche Scientifique), INSERM (Institut National de la Santé et de la Recherche Médicale), 34094 Montpellier, France; simon.george@mgx.cnrs.fr; 6 DANEC, Sangolqui/Rumiñahui, Sangolquí, Pichincha 171102, Ecuador; rpoveda@danec.com; 7 PalmElit SAS, 34980 Montferrier-sur-Lez, France; claude.louise@palmelit.com

**Keywords:** transcriptome, abscission, ripening, oil palm, reproductive development, signal transduction, hormone, cell separation, *Elaeis oleifera*

## Abstract

The developmentally programmed loss of a plant organ is called abscission. This process is characterized by the ultimate separation of adjacent cells in the abscission zone (AZ). The discovery of an American oil palm (*Elaeis oleifera*) variant that does not shed its has allowed for the study of the mechanisms of ripe fruit abscission in this species. A comparative transcriptome analysis was performed to compare the fruit AZs of the non-shedding *E. oleifera* variant to an individual of the same progeny that sheds its ripe fruit normally. The study provides evidence for widespread perturbation to gene expression in the AZ of the non-shedding variant, compared to the normal fruit-shedding control, and offers insight into abscission-related functions. Beyond the genes with known or suspected roles during organ abscission or indehiscence that were identified, a list of genes with hormone-related functions, including ethylene, jasmonic acid, abscisic acid, cytokinin and salicylic acid, in addition to reactive oxygen species (ROS) metabolism, transcriptional responses and signaling pathways, was compiled. The results also allowed a comparison between the ripe fruit abscission processes of the African and American oil palm species at the molecular level and revealed commonalities with environmental stress pathways.

## 1. Introduction

Fruit abscission is a developmentally regulated process that facilitates seed dispersal of plant species and an agronomically important character and domestication trait for crop species [1,2]. There are two species of oil palm, *Elaeis guineensis* from Africa and *Elaeis oleifera* from South and Central America, which diverged from each other approximately 7–16 million years ago [3,4]. Despite the evolutionary distance, the two species can be crossed to form hybrids, and *E. oleifera* has agronomic characters of interest for oil palm improvement in general (e.g., short growth stature and low saturated oil) [5]. We previously found differences in the abscission zone (AZ) anatomy between *E. guineensis* and *E. oleifera* [6]. While both AZs are located at the boundary area between the mesocarp and the pedicel, the *E. guineensis* AZ is made up of around 8–10 cells layers with pectin rich cell walls, and the *E. oleifera* AZ is less distinct; that creates an abrupt transition between the mesocarp and pedicel tissues [6]. While we have previously examined in great detail the ripe fruit abscission processes at the molecular and cellular levels in the multilayer AZ of the African oil palm [7,8,9,10,11], very little is known about the molecular processes of abscission in *E. oleifera* and how the abscission processes have diverged between these two closely related species separated by millions of years of evolution.

Organ abscission is a complex developmental phenomenon, regulated by environmental and physiological signals, and involves multiple signal transduction pathways, which lead to major transcriptional changes that result in cell separation in the AZ. Studies of Arabidopsis, which sheds floral organs, and tomato, which sheds flowers and fruit, have offered insight into the core mechanisms of the abscission process, key to which are hormone metabolism, signaling and transcriptional responses in the AZ [12,13,14]. The abscission process can be divided into four commonly observed stages that include (1) AZ differentiation, (2) AZ acquisition of competence to respond to abscission-inducing signals, (3) activation and execution of abscission and (4) the differentiation of the protective layer. Stage 2, the competence to respond to signals, involves multiple hormone pathways, in particular ethylene (ET). Indeed, ethylene has been widely implicated as an organ-abscission inducer in many species, including oil palm, while auxin is known to inhibit organ abscission [1,7,9,12]. The current model for the acquisition of AZ competence involves the depletion of auxin in the AZ cell layers, which appears to be mediated by an increase in ethylene biosynthesis, the production of reactive oxygen species (ROS) and a carbohydrate imbalance [15]. In addition to ethylene and auxin, other hormones are implicated in the acquisition of competence, as well as the activation and execution of abscission, including abscisic acid (ABA), jasmonic acid (JA) and cytokinins (CKs), while yet others have been shown to inhibit abscission, including gibberellins (GAs), polyamines (PA) and brassinosteroids (BRs) [12].

In the current study, we examined the fruit abscission character within a population of *E. oleifera*, in which we previously identified a variant that does not shed (non-shedding, NSd) its fruit [6]. We examine further the NSd phenotype at the anatomical and molecular levels. We first examined the AZ anatomy of the variant and found no evidence of phenotypic differences when compared to the normal individual’s AZ. Our hypothesis is that the non-shedding phenotype is not the result of the lack of an AZ but that the gene expression network is perturbed in the non-shedding variant. To test this hypothesis, we used an RNA-Seq approach to globally determine the expression of genes affected in the variant compared with that of an *E. oleifera* individual that normally sheds its fruit. The results reveal the core mechanisms of fruit abscission in *E. oleifera* and provide insight into the commonalities and divergence between this American species and the African species, *E. guineensis.*

## 2. Materials and Methods

### 2.1. Plant Material

American oil palm (*Elaeis oleifera*) fruits were collected from a palm population of 152 individuals from 18 progenies planted at the Colé station (DANEC) in Ecuador since 2006. The fruit was derived from an open pollination amongst the population. We estimated the stage of ripeness of the fruit by observing the position of the fruit bunch attachment within the whorl and based on the color of the fruit. The ripe fruit bunch was that which was positioned the lowest in the whorl of fruit bunches and had orange-colored fruit, at which time the fruit could be easily loosened and separated from the bunch. This is the typical stage at which fruit bunches are harvested for oil extraction. The fruit bunch positioned just higher than the ripe bunch in the whorl had less orange-colored fruit, and was at the pre-ripe (PR) stage, with fruit more firmly attached to the fruit bunch. 

### 2.2. Phenotyping the Abscission Character

To phenotype the abscission character of the *E. oleifera* population described above, a total of 106 in vitro tests were performed on 93 individuals from the population that allowed the calculation of the Abscission Index (AI), as previously described [6,16]. Briefly, the abscission phenotypes were defined by four classes: A, no cell separation; B, partial cell separation in primary AZ; C, extensive but not complete cell separation in primary AZ; and D, complete cell separation in primary AZ [6,16]. In the present study, we used the following formula to calculate AI:

(1) $$\mathrm{AI}=\frac{{3*n}_{A}{+1*n}_{B}+\left(-1\right){*\;n}_{C}+\left(-3\right){*\;n}_{D}}{n_{A}{+n}_{B}{+n}_{C}{+n}_{D}}$$

where n is the total number of observations per class. Negative and positive values of AI indicate a low and high level of competence of the fruit AZ to execute cell separation, respectively. For each test, approximately 24 similar fruits were collected at the ripe stage from 8–12 fruit spikelets removed from the upper-middle section of the bunch. The bases of the fruit containing the AZ were then collected, and phenotypes were determined, as described previously [6,16]. The heritability was estimated based on the data from individuals with repeated measurements (n = 12) as the ratio of the genotypic vs. phenotypic variances. The broad-sense heritability (H^2^) was calculated based on the data from individuals with repeated measurements as follows:

$$H^{2}=V_{g}/V_{p}$$

Genetic (V_g_, n = 12) and phenotypic (V_p_, n = 25) variances were calculated using var function in R software (https://www.R-project.org/, accessed on 20 June 2021).

### 2.3. RNA Extraction for Transcriptome Analyses

**Figure 1:**
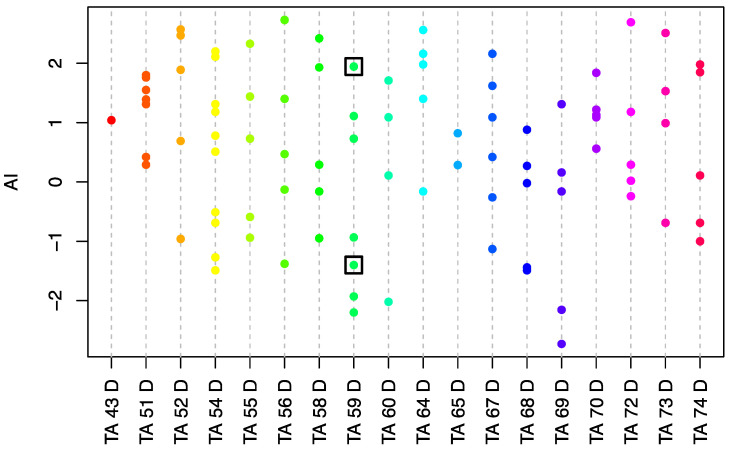
The phenotypic analysis using the in vitro test revealed a range of AI values within the Elaeis oleifera population (93 individuals phenotyped from 18 progenies). Each colored circle represents the average AI value for a single individual. Negative AI values reflect a low competence for the AZ to undergo cell separation, while positive AI values indicate a high AZ cell separation competence. The boxed green circles are the two individuals with contrasted abscission phenotypes selected for the transcriptome analysis. The green boxed circle with the negative AI is the non-shedding individual selected for the transcriptome analysis because it does not shed its fruit under field conditions, and seed germination can be observed (Figure 2).

For the transcriptome analyses, two palms within the same progeny (Figure 1, TA59D) were selected based on their contrasted AI values and contrasted abscission characters in field conditions: the palm individual 28–7, which sheds its fruit normally (Sd), and palm 26–11, that is the non-shedding oil palm variant (NSd), with which little or no fruit abscission was observed in the field. From undamaged fruit, AZs between the pedicel and the mesocarp were isolated with a scalpel from the base of each fruit and collected as previously described [11]. Three biological replications were sampled from each fruit bunch collected from each individual, for a total of twelve samples, which included three PR and three ripe Sd, as well as three PR and three ripe NSd. AZ tissues were flash frozen in liquid nitrogen, stored at −80 °C, then transported on dry ice to Montpellier. Total RNAs were extracted as described previously [17]. Construction of the twelve cDNA libraries and llumina (NovaSeq 6000) sequencing of the corresponding RNA samples was carried out by MGX platform (Montpellier Genomix, http://www.mgx.cnrs.fr/, accessed on 8 November 2019). The data discussed in this publication were deposited in NCBI’s Gene Expression Omnibus and are accessible through GEO Series accession number GSE186050.

### 2.4. Histology

For histological studies, samples were processed to obtain resin-embedded sections, which were stained with toluidine blue, then examined by light microscopy as described previously [9]. Longitudinal sections were made at the base of the fruit containing the pedicel, AZ and mesocarp from ripe fruit at similar stages of bunch development sampled from both the NSd and Sd trees of the same age. At least 8–12 fruit bases were sampled and processed for the histology analysis. At the same time that the sampling for the histology was done, fruit AZs were also sampled from the same trees for the transcriptome analysis described above.

### 2.5. Analysis of Differential Gene Expression

Reads obtained by Illumina NovaSeq sequencing (single reads, 100 cycles) were demultiplexed and trimmed using Bcl2fastq (v2.20.0.422). Total trimmed reads per sample are indicated in the Table S1. All the trimmed reads (331,907,473 total reads) passed the FastQC quality checks and were then mapped using the BWA-MEM package with default parameters [18] to a reference transcriptome derived from the genomic sequence of the African oil palm (*E. guineensis*) for which a fully annotated genome sequence is currently available [19]. The latest version of the oil palm transcriptome released by NCBI was used for mapping (http://genomsawit.mpob.gov.my; GCF_000442705.1_EG5_RNA.fna, accessed on 23 July 2019). Read counts were obtained from the resulting bam files by means of the program Samtools-idxstats [20]. For each sample, the total reads aligned (277,584,786 total reads, 84% of total trimmed reads) to reference genome is indicated in the Table S1. Counts corresponding to different transcripts of the same locus were then merged. The R-based software DESeq package (default parameters, version 1.28.1 [21] was used to identify genes displaying significantly different expression levels in the Sd and NSd palms, applying padj cutoff < 0.01 and log2 fold change (LFC) cutoff (−1 > LFC > 1). After mapping to the reference transcriptome, the number of reads per kilobase and million reads (RPKM) in each sample were then calculated.

### 2.6. Functional Annotation of Genes and Identification of Enriched Categories

Functional annotation of differentially expressed genes (DEGs) was performed using the web-based Mercator/Mapman 3.6 tool (https://mapman.gabipd.org/app/mercator, accessed on 10 June 2020) [22] and expert curation based on recent literature devoted to homologous genes characterized in model plants. To evaluate the significance of enrichment for each functional category, Fisher’s exact test was employed, with a false discovery rate (FDR) cutoff of <0.05.

## 3. Results

### 3.1. Abscission Phenotype Analysis of an E. oleifera Population

**Figure 2:**
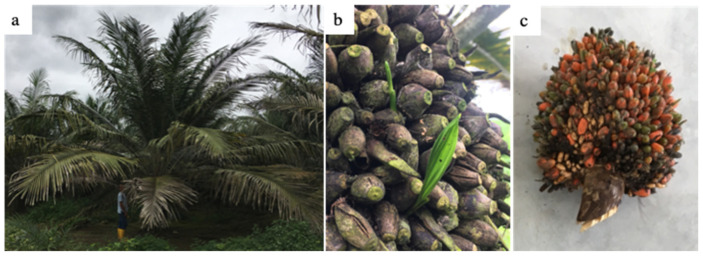
An *E. oleifera* variant was identified that does not abscise its fruit [6]. (**a**) The non-shedding *E. oleifera* palm that was examined in this study is approximately 15 years old and does not have any other notable phenotypic differences from the population used for the study. (**b**) Fruit remain attached to the fruit bunch, become dry, and eventually, seed germination can be observed. (**c**) The fruit bunch with ripe fruit from the non-shedding (NSd) palm examined in this study.

The in vitro phenotypic test was used to determine the competence to undergo cell separation in the AZ of fruits collected from the *E. oleifera* population, based on the calculation of the AI value (Figure 1). The test revealed a large range of AI values throughout the population (−2.73–2.73), and a high heritability value of 0.88 was estimated for the character. Within the population, there was one individual (palm 26–11) within the TA59D genetic background that had both a low AI (low competence for cell separation), and seed germination was observed from fruit that remained attached to the fruit bunch (Figure 2a,b). Observations in the field over a period of three years were made for this palm, and no fruit were observed to abscise or shed from this individual, which provided corroborative evidence that this palm has an NSd character. For the transcriptome analyses, fruit bunches from another individual (palm 28–7) within the same TA59D genetic background was identified with a high AI and with fruit that separate from the fruit bunch and found at the base of the palm, indicating fruit abscission and shedding occurred and is a normal Sd character.

### 3.2. The Phenotypic Comparison of Fruit-Shedding and Non-Shedding Oleifera Palms

**Figure 3:**
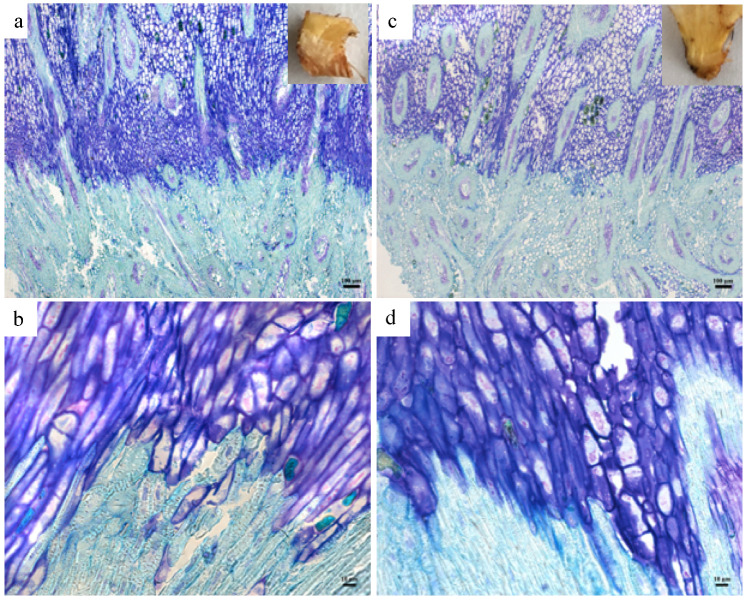
Comparison of the shedding and non-shedding fruit bases. (**a**) Base of fruit of normal shedding fruit. (**b**) Higher magnification of the fruit base from a normal shedding fruit. (**c**) Fruit base from the non-shedding fruit. (**d**) Higher magnification of base of fruit from a non-shedding fruit. Longitudinal sections were stained with toluidine blue. Mesocarp cells situated above the pedicel stained darker blue. The photos shown are representative of at least 6 replicate fruit bases examined.

The NSd character could be due to a developmental defect that results in a non-functional AZ, such as those observed for the *jointless* mutant of tomato and the *BLADE-ON-PETIOLE* double mutant *bop1 bop2* of Arabidopsis [23,24]. To determine whether the AZ has a developmental defect, a histological analysis was performed. The fruit bases of the NSd palm were compared to the normal Sd palm that were prepared for the transcriptome analyses (Figure 3). The results suggest that the NSd character is not caused by a defect in the AZ anatomy and is more likely caused by a perturbation of the gene network that controls the competence to respond to the abscission signals or changes to the cell wall composition that could block fruit from undergoing cell separation in the AZ.

### 3.3. Gene Expression Profiling of AZ Samples from Normal Fruit-Shedding and Non-Shedding Variant Oleifera Palm

RNA-Seq was performed with AZ total RNA extracted from the two contrasting NSd and Sd oleifera palms and at two stages of development, PR and ripe. A total of 277,584,786 trimmed reads aligned to 29,040 loci. A total of 24,373 loci that had a minimum of 1 RPKM were used for the analysis. First, differential expression was studied in detail by the identification of genes showing either up- or downregulation in AZ of the PR Sd compared to the ripe Sd, which could be easily loosened and separated from the bunch. We presumed that genes either upregulated or downregulated between these two stages of development have potential functions during the abscission process of ripe fruit. A total of 875 DEGs (padj < 0.01; LFC ± 1) were identified from this step. Second, differential expression was studied in detail by the identification of genes showing either up- or downregulation in AZ of the ripe NSd compared to the ripe Sd. A total of 1146 DEGs (padj < 0.01; LFC ± 1) between normal Sd and variant ripe AZs were identified. Then, we selected DEGs that were common between those two lists of DEGs. A total of 274 DEGs were identified, 172 of which were more highly expressed in the NSd AZ (genes classified as NSd up) and 102 DEGs lower in the NSd (genes classified as NSd down) (Table S2). DEGs with an RPKM amount less than 20 were eliminated, which resulted in a final list of 139 DEGs: 63 NSd up- and 76 NSd downregulated DEGs in the NSd AZ. The DEGs are listed in Table S3, along with their orthologs from Arabidopsis and publicly available annotations and information on phenotypes of corresponding mutants.

### 3.4. Functional Annotation of Differentially Expressed Genes and Identification of Enriched Categories

**Figure 4:**
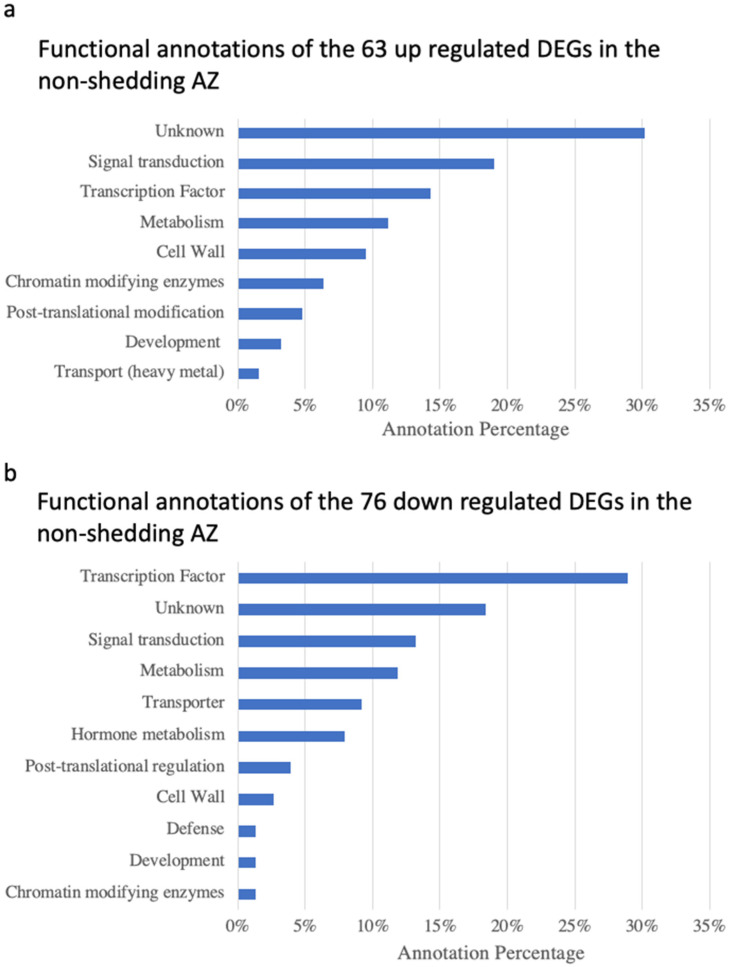
Functional annotations of the up- (**a**) and downregulated (**b**) DEGs revealed in the NSd fruit palm AZ compared with the Sd fruit AZ.

In order to investigate the biological significance of differential gene expression patterns observed between Sd and NSd palms, the Mercator/Mapman annotation system was used to assign functional categories to each of the 139 DEGs (Figure 4). Amongst the 63 NSd up DEGs, the highest number had unknown (30%) annotations, followed by annotations related to signal transduction (19%), transcription factors (TFs, 14%), metabolism (11%), cell wall (10%), and annotations related to chromatin modification, transport, post-translational modification and development of less than 10%. Of the 76 NSd down DEGs, the highest percentage was for TFs (29%); 18% were unknown; other annotations related to signal transduction (13%); metabolism (12%); while annotations related to transport, hormone metabolism, post-translational regulation, cell wall, defense, development and chromatin modification all had less than 10%.

The percentage representation of each gene functional category in the reference genome, from which the mapped transcriptome was derived, was compared to the corresponding percentage representations of genes of the same category within the 139 DEGs. In this way, we were able to test for significant enrichment of each functional category within both the up and down NSd DEG sets. We detected one instance of significantly enriched gene categories (Fisher’s exact test with FDR cutoff < 0.05) related to hormone metabolism.

### 3.5. Comparison with E. guineensis Ripe Fruit AZ Transcriptome

To compare *E. oleifera* fruit abscission with what is known about ripe fruit abscission in *E. guineensis*, we compared the DEG list identified in the current study with those found in a recent transcriptome study of *E. guineensis* [11]. In total, there were six DEGs found in common from the two analyses (Table 1).

## 4. Discussion

### 4.1. Evidence for Transcription Factors and Hormone-Signaling Pathways Common to Organ Abscission Processes That Function during Oil Palm Fruit Abscission

**Figure 5:**
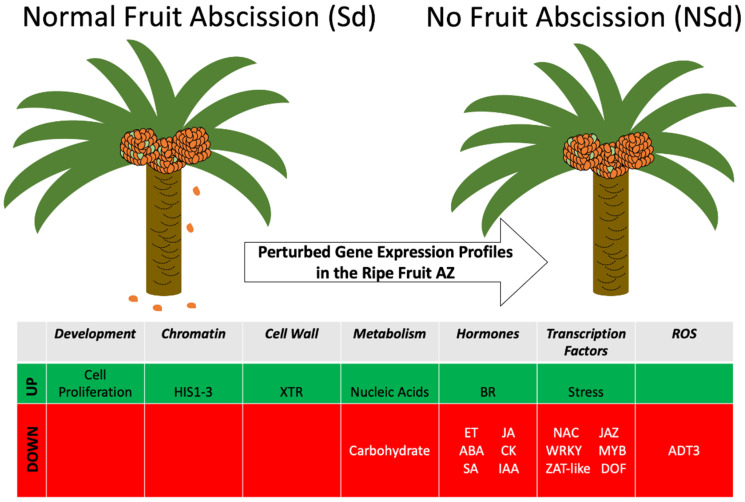
The non-abscission phenotype (NSd) of the *E. oleifera* palm is a result of both up- and downregulated gene expression in the ripe fruit variant AZ. When fruit are ripe, they abscise and fall to the ground and are found around the base of the normal palm, while no fruit is found at the base of the variant that does not undergo abscission. Green signifies globally higher, while red indicates lower transcript amounts in the NSd variant. DOF (Dof-type zinc finger DNA-binding family protein), NAC, (NAM, ATAF and CUC family member), JAZ (Jasmonate-Zim-domain protein), ADT3 (AROGENATE DEHYDRATASE 3), HIS1-3 (Histone H1-3), XTR (Xyloglucan endotransglycosylase-related protein) brassinosteroid (BR), Indole-3-acetic acid (IAA), ethylene (ET), jasmonate (JA), abscisic acid (ABA), cytokinin (CK), salicylic acid (SA).

The current study provides evidence that the NSd fruit character identified within an *E. oleifera* population is caused by perturbations to gene expression profiles, and hence, the gene network that underlies the abscission process. A histological comparison of the NSd and Sd fruit AZs revealed no apparent differences, while the transcriptome study provided substantial evidence for changes in gene expression in the ripe fruit NSd AZ compared to a normal Sd fruit AZ, directly or indirectly related to the inability of this individual to shed fruit. The results indicated no obvious difference in the AZ anatomy between the two palms. As previously shown, the AZ of *E. oleifera* is different from that of *E. guineensis* [6]. Both the NSd and Sd palms had similar characteristics, which included an abrupt transition between the mesocarp and the pedicel, with a mesocarp that stains darker blue than the pedicel. Studies with tomato and Arabidopsis have identified mutants that are affected in AZ development, all of which affect transcription factors, which confer a non-abscission phenotype due to defects in AZ development [23,24]. In contrast, a number of mutants, in particular, those of the model Arabidopsis, have been identified that provide insight into the AZ acquisition of competence to respond to abscission signals during floral organ abscission, which includes mutations that affect hormone signaling, transcriptional response and other signaling pathways [12]. In our current study, the most common annotation for both up- and down-missregulated DEGs in the non-shedding variant are for genes that encode functions of transcription factors and signal transduction (Figure 4). Furthermore, an enrichment test found gene annotations for hormone metabolism significantly more represented in DEGs. These results suggest that the NSd fruit character is caused by perturbations to the expression of genes that encode for functions involved in the transcriptional regulation and signal transduction events important for fruit shedding to take place, in particular, those related to hormones, as highlighted in previous transcriptome studies [6,33,34,35,36,37,38]. Notably, several genes with downregulated profiles encode for functions that involve plant hormone transcriptional regulation, signaling and homeostasis, including those for ethylene (ET), jasmonic acid (JA), abscisic acid (ABA), cytokinin (CK), salicylic acid (SA) and auxin (Figure 5). Genes related to ethylene and auxin signaling, homeostasis and response were found both up- and downregulated in the NSd variant. Our hypothesis is that the deregulation of these important hormone-related genes results in an AZ incompetent to respond to abscission signals [39,40,41,42].

Within the group of genes with perturbed gene expression, key genes were found that are implicated in the cell-separation processes of Arabidopsis and could provide insight into the basis of the NSd phenotype of the variant palm. Firstly, a DEG (LOC105043978) that encodes a protein similar to the Arabidopsis Dof-type zinc finger DNA-binding family protein/CYCLING DOF FACTOR 4 (CDF4) induces a number of key floral abscission-related processes in Arabidopsis [43]. The CDF4 TF increases endogenous ABA levels through the upregulated transcription of the ABA biosynthesis genes 9-cis-epoxycarotenoid dioxygenase 2, 3 (NCED2, 3) and increases ROS by the suppression of H_2_O_2_ scavenging through the repressed expression of the *catalase2* (*CAT2*) gene. Additionally, CDF4 activates the *PGAZAT* gene, which encodes a key polygalacturonase (PG) involved in floral organ abscission [44]. The reduced expression of a *CDF4-like* gene in the ripe fruit AZ of the NSd variant could result in the inhibition of these key abscission processes and a potential basis of the NSd phenotype. Further evidence of perturbations to ABA signaling and response comes from the identification of two DEGs (LOC105060191 and LOC105051963) that encode a protein similar to a WRKY DNA-binding protein40 (WRKY40), which is a central negative regulator of ABA-related signal transduction [45]. ABA appears to repress the WRKY40 transcription repressor during stressful conditions, in cooperation with light, to allow plants to adapt to environmental stresses [46]. In addition to ABA signal transduction, there is a DEG (LOC105047868) for the ABA transporter gene *AtABCG40* [47] that is also less expressed in the NSd variant AZ, suggesting transcriptional changes for ABA homeostasis and signal transduction are both affected.

The second notable DEG linked to an Arabidopsis cell-separation process encodes a protein similar to DELAYED DEHISCENCE 2 (DDE2), an allene oxide synthase (AOS), which is a key enzyme in JA biosynthesis [48]. The dde2-2 mutant, a result of a frameshift in the gene, is defective in anther dehiscence [49]. The reduced expression of an *AOS* gene in the NSd variant palm AZ could lower JA amounts, whereas JA is a positive inducer of both Arabidopsis anther dehiscence and floral organ abscission [50]. Indeed, the current study found five other JA-related transcripts with perturbed expression profiles in the NSd variant palm fruit AZ. Importantly, a number of downregulated DEGs are transcripts that encode JA ZIM-domain proteins (JAZ), which are repressor proteins of the JA response [48]. A total of four DEGs (LOC105042036, LOC105051062, LOC105053062 and LOC105058608) encode JAZ-like proteins, JAZ1, JAZ2, JAZ10 and JAZ2/TIFY10B, shown to function as repressors of the Arabidopsis JA response [51,52,53]. JA is known to accumulate in response and function to resist abiotic or biotic stresses but also has roles during development of roots, reproductive organs and plant senescence [48]. A major control point of the JA response is when JA or its amino acid derivatives (e.g., JA-isoleucine) bind to the JA receptor (CORONATINE INSENSITIVE1), which results in the degradation of the JAZ repressor proteins via the ubiquitination-proteasome proteolytic pathway and releases the inhibition of the basic helix-loop-helix(bHLH) TF protein MYC2, a master regulator of the JA transcriptional response [48]. In our study, it is remarkable to find four different gene loci, all with corresponding transcripts that normally increase from the PR stage to the R stage in a normal fruit-shedding palm, that have lower accumulation in the NSd variant AZ. Finally, there is an additional DEG (LOC105047615) that encodes a protein similar to the bHLH TF JASMONATE-ASSOCIATED MYC2-LIKE1 (JAM2) that also negatively regulates JA responses and which is mostly antagonistic to the positive activity of MYC2 in JA response [54]. Collectively, these results provide substantial evidence for major perturbations in key components of JA-related signaling and biosynthesis, which could be a basis of the NSd phenotype.

Ethylene and auxin are antagonistic with key abscission regulatory roles, with ET that promotes and auxin that inhibits the abscission process. Furthermore, an ET-dependent decrease in auxin in the AZ leads to the acquisition of ET sensitivity and abscission competence [15,39]. Previous work has shown that ET can accelerate *E. guineensis* fruit abscission through modulation of gene expression [7,9,11]. In the current study, the NSd variant had reduced amounts of two similar DEGs (LOC105043735 and LOC105053951) for the ERF-1 (Ethylene Response Factor-1) that functions to maintain a balance between cambium-driven growth and adaptation to environmental stress [55]. This is notable, given the similarities between the molecular basis of meristem maintenance and the abscission process and the importance of the environment in modulating the acquisition of AZ competence and function [14,16,56,57]. In contrast, other DEGs (LOC105059334, LOC105054588, LOC105060890, LOC105050553, LOC105035740, LOC105043386, LOC105043733) for ERF-related TF genes are upregulated in the NSd variant, the majority of which are related to abiotic and biotic stress [58,59,60,61,62,63] and one (LOC105042035) for fruit ripening [64]. Finally, there is one DEG (LOC105053311) that encodes LHT1, involved in the uptake of ACC, the precursor of ET [32], which has lower expression in the ripe fruit NSd AZ than in the normal Sd AZ. This suggests the NSd variant not only sustains changes to ET related transcriptional responses but also to ET homeostasis, which could also be a basis for the lack of competence to undergo cell separation in the NSd AZ.

Auxin depletion in AZ cells, which can be a result of different mechanisms, including auxin metabolism (biosynthesis, conjugation and degradation) and cellular auxin transport, results in an increase in ET sensitivity that induces cell separation and organ abscission [15]. In the current study, we found four auxin-related DEGs with perturbed gene expression in the variant AZ relevant to auxin homeostasis and response. Three DEGs that encode proteins involved in auxin transport were identified to be less expressed in the variant ripe fruit AZ, one (LOC105055275) for an auxin efflux carrier family protein similar to PIN-LIKES 5 (PILS5), which regulates intracellular auxin homeostasis [65], one (LOC105037126) for a protein similar to IAR3, which hydrolyzes an inactive conjugated form of auxin (IAA-alanine) and releases bioactive auxin (IAA) [66], and one (LOC105038143) for a calcineurin B-like protein (CBL)-interacting protein kinase (CIPK) involved in basipetal auxin transport [67]. In contrast, a DEG (LOC105052677) that encodes a protein similar to PINOID (PID)-binding protein, involved in auxin signal transduction and response [68], was significantly higher in both the PR and ripe variant fruit AZ. These results provide evidence for major transcriptional changes that may lead to both changes in auxin homeostasis and signaling in the NSd variant and another possible mechanism for the lack of competence of the NSd AZ to undergo abscission.

### 4.2. Evidence That Changes in ROS Homeostasis and Hemicellulose Metabolism Are a Part of the Basis for the NSd Phenotype

Other evidence for transcriptional perturbations related to hormone homeostasis include two DEGs (LOC105036203 and LOC105041939) that encode proteins similar to a UDP-glucosyl transferase 85A2 (UGT85A2) implicated in CK homeostasis [69] and one (LOC105036760) for an SA 5-hydroxylase (S5H/DMR6) implicated in SA homeostasis [70], both of which are expressed lower in the ripe variant fruit AZ. Finally, two DEGs (LOC105056795 and LOC105056794) for protein similar to a phosphate-responsive 1 family protein EXORDIUM (EXO), involved in signaling processes that coordinate BR responses with environmental or developmental signals [71], was expressed higher in the ripe fruit variant AZ. A recent article showed that exogenous BR application reduced ethylene-induced litchi fruitlet abscission, caused by a lower ET production and the suppression of ethylene biosynthetic gene expression [72]. While in our study, we found no evidence for a repression of ET biosynthesis, the variant has perturbed transcription of BR signaling.

ROS production is involved in the abscission of different organs, including Arabidopsis floral organs, lupine flowers, cotton leaves, water-deficit-stress-induced cassava leaves and carbohydrate-stress-induced longan fruit abscission [73,74,75,76,77,78]. During Arabidopsis floral organ abscission, differential ROS distribution characterizes the AZ residuum cells (RECs), which have a higher accumulation of superoxide (O_2_^−^) and secession cells (SECs), with a higher accumulation of hydrogen peroxide (H_2_O_2_). Cell separation occurs between the RECs and the SECs, while RECs remain attached to the plant and eventually form the protective layer, and SECs are shed with the floral organ. The accumulation of H_2_O_2_ is related to a higher amount of lignin formation in the SECs, which may limit the diffusion of cell wall enzymes, while lignin is absent in the RECs cell walls that are more accessible to enzymatic activity [74,75]. During carbohydrate-starvation-induced longan fruit abscission, a burst of ROS in the AZ, in particular, H_2_O_2_, is compartmentalized differentially and could function both in cell-wall modifications and as a plant-signaling molecule [78]. Indeed, the ROS pathway can be localized in different cellular compartments and functions during development through interactions with different phytohormones, including ET, JA, SA, ABA and GA [79]. In the current study, we identified 4 DEGs related to ROS homeostasis with decreased expression in the AZ of the NSd variant, including one (LOC105054147) for the phenylalanine biosynthetic enzyme ADT3, which buffers excess ROS during seedling development [80], one (LOC105043978) for the TF CDF4, which suppresses H_2_O_2_ scavenging leading to abscission [43], one (LOC105053223) for plantacyanin, involved in anther dehiscence [81], one (LOC105036974) for a peroxidase, a potential antioxidant enzyme, and one (LOC105046069) for an NAC TF shown to be involved in the induction of H_2_O_2_/ROS production [82]. Taken together, the data provide substantial evidence for major changes in ROS-related transcriptional activity in the NSd variant AZ, which could result in changes to ROS homeostasis in the AZ and be a potential basis of the NSd phenotype.

The most notable DEGs with increased expression in the NSd variant are five (LOC105049189, LOC105050826, LOC105037481, LOC105049188 and LOC105049227) that encode for xyloglucan endotransglycosylase-related proteins (XTRs), also known as xyloglucan endotransglucosylase/hydrolases (XTHs). XTHs can have either xyloglucan endotransglucosylase (XET) or xyloglucan endohydrolase (XEH) activities and are involved in the modification of xyloglucan, the most abundant hemicellulose in the primary cell wall, which forms crosslinks between cellulose microfibrils [83,84]. XTHs have roles at different stages of plant growth and development and in response to biotic and abiotic stresses and are also implicated in litchi fruitlet abscission, where their genes are activated by ethylene [84,85,86]. Furthermore, ectopic expression of the litchi *XTH19* in Arabidopsis resulted in precocious floral organ abscission [86]. In the current study of *E. oleifera*, four of the XTR DEGs correspond to the loci most similar to the Arabidopsis gene XTR6/XTH23 (AT4G25810) involved in lateral root development during salt stress [87]. While our basic histological studies did not detect changes in the AZ anatomy that could explain the NSd phenotype (Figure 3), modifications of the cell-wall composition could also be a basis of the NSd phenotype. Future studies will examine in more detail the cell wall structure in these variants to determine whether the transcriptional changes related to these XTR genes we observed in the current study result in changes in cell-wall composition, in particular, to the hemicellulose xyloglucan fraction.

### 4.3. A Comparison of E. oleifera and E. guineensis Suggests Abscission Processes Related to Environmental Stress and Epigenetic Modifications

This study also provides a unique opportunity to compare the molecular basis of ripe fruit abscission of the two species of oil palm, *E. guineensis* from Africa and *E. oleifera* from South and Central America. Notably, a total of six DEGs identified in the current study of *E. oleifera* were common to those found in the previous study of *E. guineensis* [11]. These DEGs are particularly relevant, given the differences in the criteria used for screening for DEG candidates in the two studies: the use of a non-shedding individual for the *E. oleifera* study and the use of a large-scale, multifaceted analysis of ripe fruit AZ induced by ethylene with *E. guineensis* [11]. A closer look at the DEGs identified is warranted. One DEG (LOC105043733) encodes a member of the ERF (ethylene response factor)/AP2 TF family, with a sequence most similar to the Arabidopsis ERF104, which is involved in response to cold stress [25,26]. In the two species of oil palm, expression of *ERF104* decreases during development prior to abscission and appears to decrease during the ethylene treatments of *E. guineensis* [11]. Another candidate (LOC105059363) encodes a structurally divergent linker, histone H1 (HIS1), most similar to Arabidopsis HIS1-3, whose gene is induced in response to drought [27]. In the two species of oil palm, expression of *HIS1-3* decreases during development prior to abscission and appears to decrease during the ethylene treatments of *E. guineensis* [11]. Another candidate (LOC105060420) encodes a late embryogenesis-abundant, (LEA) hydroxyproline-rich glycoprotein, YELLOW-LEAF-SPECIFIC9 (YLS9), whose gene is induced in response to salt stress, in senescent leaves and during the hypersensitive response [28,29,30]. Expression of *YLS9* in *E. guineensis* increases during development and is highest at the stage of abscission [11]. In *E. oleifera*, expression is higher in the NSd AZ than the Sd AZ at both PR and ripe stages. Another candidate (LOC105033708) is most similar to Arabidopsis TF nonsymbiotic hemoglobin (HB1/GLB1), involved in nitric oxide (NO) homeostasis and in response to desiccation through interactions with abscisic acid (ABA). In the two species of oil palm, expression of the *HB1* increases during development prior to abscission and appears to decrease during the ethylene treatments of *E. guineensis* [11]. Another candidate (LOC105044389) encodes a protein most similar to the Arabidopsis telomere repeat binding factor-like (TRFL2), with DNA-binding domains of the SANT/myb-like family. No functional information is known from Arabidopsis about this gene, but in both oil palm species, expression increases during ripening and the stage of abscission, and it is lower in the NSd palm AZ than the Sd. Finally, the candidate (LOC105053311) encodes a lysine-histidine transporter (LHT) most similar to LHT1 found to be involved in 1-Aminocyclopropane-1-carboxylic acid (ACC) transport and ACC-induced ethylene responses of Arabidopsis [31]. Expression increases in both species during ripening and reaches a peak at the stage of abscission, while it is lower in the ripe NSd than the Sd AZ [11].

Interestingly, the majority of the DEGs common between the transcriptome studies performed with *E. guineensis* have annotations of proteins involved in the response to different stresses, including cold (*ERF104*), drought (*HIS1-3*), salt and hypersensitivity (*YLS9/NHL10*), and desiccation *HB1/GLB1* [11]. This observation corroborates well the molecular pathway similarities found between abiotic and biotic stresses and abscission [57]. Furthermore, another AZ DEG common to *E. guineensis* and *E. oleifera* is similar to a transporter of ACC involved in ACC-induced ethylene responses in *A. thaliana*, emphasizing the common role for ET in both species. Whereas we do not know the molecular basis of the NSd phenotype, the results provide evidence for changes in multiple pathways in the AZ, which suggests genome-wide changes to gene expression. To have such wide-ranging effects on gene expression, one possibility is for an epigenetic basis of the phenotype, which could be caused by chromatin modifications. Indeed, in both the *E. guineensis* and *E. oleifera* AZs, the *HIS1-3* gene was identified in our screens for genes related to the abscission processes. Furthermore, in *E. oleifera*, four different DEGs for *HIS1* were identified, all with higher expression in the ripe fruit AZ. Interestingly, H1.3 is required for DNA methylation associated with environmental stress [88]. Taking this into account with all the other stress-related DEGs found to be deregulated in the NSd AZ, the NSd phenotype appears to be related to major deregulation of genes associated with environmental stresses, which may occur early during reproductive development, with consequences on abscission timing [16].

Finally, we did not find any PG genes in the current study, which contrasts greatly to the discoveries in our previous transcriptome studies with *E. guineensis* [7,11]. In those previous studies, we identified three PG genes, *EgPG4*, *EgPGAZ1* and *EgPGAZ2*, with expression in the AZ that corresponds to the timing of abscission. While *EgPG4* is expressed in both the AZ and the mesocarp, *EgPGAZ1* and *EgPGAZ2* are expressed specifically in the AZ. Given the structural differences between the AZ of *E. oleifera* and *E. guineensis*, this may not be too surprising. While the *E. guineensis* AZ is rich in pectin and consists of multiple cell layers, the AZ of *E. oleifera* is less pronounced, with a more abrupt transition between the pedicel and the mesocarp, and possibly much less pectin-rich [6,9].

## 5. Conclusions

The current AZ transcriptome study of a non-fruit-shedding *E. oleifera* palm has allowed for detailed insight into the potential pathways that are perturbed and which may form the molecular basis of the NSd phenotype. Importantly, we identified genes and pathways affected shown previously to be relevant for organ abscission processes, in particular, hormone homeostasis, signaling and response. In particular, we found major transcriptional perturbations to factors involved in JA signaling and response and ROS homeostasis, all consistent with the lack of competence for the NSd AZ cells to undergo cell separation and shed ripe fruit. Furthermore, we also proved evidence for transcriptional perturbations related to the modification of xyloglucan, an abundant hemicellulose in the primary cell wall, which could be the structural basis for the inability of ripe fruit to detach from the fruit bunch. In the current study, we focused on one individual with the most dramatic abscission phenotype, which is unable to shed its fruit naturally in field conditions. Future studies could examine the other individuals of the *E. oleifera* population that have less dramatic phenotypes to determine whether the same pathways are affected. Furthermore, based on both the large variation observed in the population and the high heritability estimated for the character, a genetic approach to identify QTLs is promising.

## Figures and Tables

**Table 1:** Gene candidates identified in the current study that were also found in the previous transcriptome study with *E. guineensis* [11].

Eg Locus	TAIR9 Accession	Best Manual Annotation	Gene Name	References	Related Function
LOC105043733	AT5G51190	ERF (ethylene response factor 105) subfamily B-3 of ERF/AP2 transcription factor family	*ERF104*	[25,26]	Response to cold stress
LOC105059363	AT2G18050	Histone H1-3	*HIS1-3*	[27]	Response to drought stress; senescent leaves
LOC105060420	AT2G35980	Late embryogenesis abundant (LEA)/YELLOW-LEAF-SPECIFIC GENE 9	*YLS9/NHL10*	[28,29,30]	Response to salt stress; senescent leaves; hypersensitive response
LOC105033708	AT2G16060	Nonsymbiotic hemoglobin 1	*HB1/GLB1*	[31]	Nitric oxide (NO) homeostasis and interacts with ABA processes of plants (e.g., response to desiccation)
LOC105044389	AT1G07540	Telomere-binding protein	*TRFL2*	-	-
LOC105053311	AT5G40780	Lysine histidine transporter 1	*LHT1*	[32]	Transport of ACC and ACC-induced ethylene responses in A. thaliana

## Data Availability

The data discussed in this publication were deposited in NCBI’s Gene Expression Omnibus and are accessible through GEO Series accession number GSE186050.

## Supplementary Materials

The following are available online at https://www.mdpi.com/article/10.3390/genes12111724/s1, Table S1: RNA-Seq overview, Table S2: 274 DEGs (UP and DOWN), Table S3: 139 DEGs (UP and DOWN).
